# Public perceptions, individual characteristics, and preventive behaviors for COVID-19 in six countries: a cross-sectional study

**DOI:** 10.1186/s12199-021-00952-2

**Published:** 2021-03-03

**Authors:** Ryosuke Fujii, Kensuke Suzuki, Junichiro Niimi

**Affiliations:** 1 Department of Preventive Medical Sciences, Fujita Health University School of Medical Sciences, 1-98 Dengakugakubo, Kutsukake-cho, Toyoake, 470-1192 Japan; 2 Department of Economics, The Pennsylvania State University, University Park, PA 16802 USA; 3 Department of Business Management, Meijo University, 1-501 Shiogamaguchi, Tempaku-ku, Nagoya, 468-8502 Japan

**Keywords:** COVID-19, Public perception, Preventive behavior, Wearing a mask, Handwashing, Avoiding social gatherings

## Abstract

**Background:**

Public perceptions and personal characteristics are heterogeneous between countries and subgroups, which may have different impacts on health-protective behaviors during the coronavirus disease 2019 (COVID-19) pandemic. To assess whether self-reported perceptions of COVID-19 and personal characteristics are associated with protective behaviors among general adults and to compare patterns in six different countries.

**Methods:**

This cross-sectional study uses the secondary data collected through an online survey between 15 and 23 April 2020 across six countries (China, Italy, Japan, Korea, the UK, and the USA). A total of 5945 adults aged 18 years or older were eligible for our analysis. A logistic regression model was used to estimate odds ratios (OR) and 95% confidence intervals (95%CI) of three recommended behaviors (wearing a mask, handwashing, and avoiding social gatherings).

**Results:**

In most countries except for China, the participants who perceived wearing a mask as being extremely effective to curtail the pandemic were more likely to wear a mask (OR, 95%CI: Italy: 4.14, 2.08–8.02; Japan: 3.59, 1.75–7.30; Korea: 7.89, 1.91–31.63: UK: 9.23, 5.14–17.31; USA: 4.81, 2.61–8.92). Those who perceived that handwashing was extremely effective had higher ORs of this preventive behavior (OR, 95%CI: Italy: 16.39, 3.56–70.18; Japan: 12.24, 4.03–37.35; Korea: 12.41, 2.02–76.39; UK: 18.04, 2.60–152.78; USA: 10.56, 2.21–44.32). The participants who perceived avoiding social gathering as being extremely effective to curtail the pandemic were more likely to take this type of preventive behavior (OR, 95%CI: China: 3.79, 1.28–10.23; Korea: 6.18, 1.77–20.60; UK: 4.45, 1.63–11.63; USA: 4.34, 1.84–9.95). The associations between personal characteristics, living environment, psychological status, and preventive behaviors varied across different countries. Individuals who changed their behavior because of recommendations from doctors/public health officials were more likely to take preventive behaviors in many countries.

**Conclusions:**

These findings suggest that higher perceived effectiveness may be a common factor to encourage preventive behaviors in response to the COVID-19 pandemic. These results may provide a better understanding of the homogeneity and heterogeneity of factors related to preventive behaviors and improve public health policies in various countries and groups.

**Supplementary Information:**

The online version contains supplementary material available at 10.1186/s12199-021-00952-2.

## Background

In December 2019, an outbreak of coronavirus disease 2019 (COVID-19) was identified in Wuhan, China. From mid-January to February 2020, the virus spread to Asian countries, and over the next few months, around the world [1]. As of 14 January 2021, a total of 92,888,380 cases and 1989,349 deaths due to the COVID-19 had been reported around the world [2]. The spread of the pandemic over time has varied substantially across not only countries but also regions within countries. Many plausible reasons underlie this geographic variation, such as the prevalence of preexisting conditions [3, 4], administrative orders, and recommendations for slowing the spread [5, 6].

Given the lack of effective pharmaceutical interventions against COVID-19, preventive measures at the individual level have been crucial to reduce the risk of infection. In fact, some recent papers have suggested that wearing a mask and handwashing are effective to prevent the spread of severe acute respiratory syndrome-coronavirus-2 (SARS-CoV-2) [7–9]. Many previous interdisciplinary studies have attempted to comprehend the determinants of individual preventive behaviors using established conceptual frameworks, e.g., the Health Belief Model (HBM). According to these studies, engaging in preventive behaviors may be triggered by a complex combination of constructs, such as barriers, optimistic biases, social contexts, science communication, and personal perceptions/beliefs [10–14]. In addition, diverse factors underlying individual perceptions, such as demographic, psychological, and socioeconomic characteristics, need to be considered [15].

Since the outbreak of COVID-19, a growing number of studies have attempted to predict the spread of the disease using mathematical modeling [16–18]. In those studies, researchers set parameters that govern individual behavior and the transmission of the disease among populations and impact the simulation results substantially. Therefore, understanding the interactions between individual demographic and socioeconomic characteristics, cultural and social contexts, perceptions about the disease, and health preventive behaviors is a fundamental step to develop advanced modeling and provide critical insights into how public health experts should best respond to the pandemic.

Given that factors related to preventive behaviors are country-dependent, in this cross-sectional study, we aimed to describe international differences in personal characteristics, cultural backgrounds, and perceptions about the pandemic, and to examine the associations between these factors and health preventive behaviors in six countries.

## Methods

### Cross-sectional online survey

In order to analyze the relationship between public perceptions, individual characteristics, and preventive behaviors for COVID-19 in six countries, this cross-sectional study was a secondary analysis using information obtained through an online survey conducted across six different countries (China, Italy, Japan, Korea, the UK, and the USA) from 15 to 23 April 2020 [19]. The online survey was performed with the support of two market research companies (Lucid Holdings, LLC, New Orleans, LA, and dataSpring, Inc., Tokyo, Japan; the data are publicly available at https://osf.io/aubkc/). The interview form was developed for this study and is available as Additional file 1 in the English version. Before participating in the online survey, informed consent was obtained from all participants after specifying that anonymized individual-level data would be made public. Participation was remunerated according to general compensation schemes defined by each company. The median response time to complete the questionnaire was about 14 min. To ensure the reliability of the survey, those who responded at less than 50% of the median response time were excluded beforehand. The original study protocol was approved by the ethics board at the University of Exeter (eUEBS003014 v2.0), covering surveys in all countries in the midst of a time-sensitive crisis. In addition, the protocol was officially reviewed by the ethics review board of Fujita Health University (HM20-182).

### Study population

A total of 6089 adults aged 18 years or older were eligible for this survey. As described in [19], the participants are confirmed to be nationally representative in terms of age structure, sex, and household income in each country. We excluded 144 individuals from the analysis because of missing values on the questionnaire, resulting in a data set consisting of 5945 individuals (2900 men and 3045 women).

### Public perceptions

Perceived susceptibility was assessed according to whether participants believed that they are or had been infected with SARS-CoV-2. Perceived severity was assessed according to whether participants believed that they would develop a serious illness requiring hospitalization because of the virus. These question items were answered in terms of percentage points (0–100%). The perceived effectiveness of each preventive behavior was assessed according to whether participants believed that a specific action would help slow the spread of COVID-19 based on the following five options: (1) not effective at all, (2) slightly effective, (3) moderately effective, (4) very effective, or (5) extremely effective. The original questions and items in the survey regarding public perceptions are summarized in the upper part of Table 1.

**Table 1:** List of variables for public perception and preventive behaviors in this study (compared with the questions and values in) and the original questionnaire

Variable name in this study	Original survey question	Values in the original survey	Values in this study
Perceived susceptibility	What do you think is the probability that you are or have been infected with COVID-19?	Probability in percentage points % (integers 0–100)	Same as questionnaire
Perceived severity	What do you think is the probability that an infected person develops a serious illness that requires hospitalization?	Probability in percentage points % (integers 0–100)	Same as questionnaire
	How effective do you believe each of these measures is in reducing the spread of the epidemic?		
Perceived effectiveness of wearing a mask	Requiring masks to be worn outside by everyone	1 = Not effective at all, 2 = Slightly effective, 3 = Moderately effective, 4 = Very effective, 5 = Extremely effective	Same as questionnaire
Perceived effectiveness of handwashing	Hand washing		
Perceived effectiveness of avoiding social gatherings	Forbidding mass gatherings		
	For each of the following behaviors, please tell us how often you engaged in them NOW		
Wearing a mask	Wear a mask	1 = Never, 2 = Rarely, 3 = Sometimes, 4 = Very often, 5 = Always	1 or 2: NOT taking preventive behaviors 3, 4, or 5: taking preventive behaviors
Handwashing	Wash your hands with water and soap, or use hand sanitizer		
Avoiding social gatherings	Participate in a social gathering with more than 20 people		

^a^The detail information of this questionnaire is available in https://osf.io/3pxkr/

### Preventive behaviors

Participants were asked questions regarding the frequency of various daily behaviors. The questions about each behavior were prefaced by the statement, “Please tell us how often you have engaged in each of the following behaviors.” The participants responded to the question items about these behaviors on a five-point scale (never, rarely, sometimes, very often, or always). We selected two behaviors that had been recommended by government offices and national official institutes: wearing a mask and either handwashing with water and soap or using hand sanitizer. The participants who answered “never” or “rarely” were defined as those who were *not* taking preventive behaviors, whereas those who answered “sometimes,” “very often,” or “always” were defined as those who *were* taking preventive behaviors. By contrast, we selected one unfavorable behavior—participation in a social gathering with more than 20 people—for which the participants who answered “never” or “rarely” were defined as those who *were* taking preventive behaviors, whereas those who answered “sometimes,” “very often,” or “always” were defined as those who were *not* taking preventive behaviors. Participants were also asked questions regarding the different reasons why they changed their daily behaviors. In this study, we selected the variables of recommendations from family/friends, doctors/public health officials, or politicians, and conformity. Since the questionnaire did not specify the changed behavior, the responses to those questions should be interpreted as representing how likely an individual’s behavior was affected by conformity or recommendations from others during the pandemic. The questions and items regarding health behaviors are summarized in the bottom part of Table 1.

### Statistical analysis

We calculated means and standard deviations for continuous variables and numbers and percentages for categorical variables. We performed country-stratified logistic regression analyses to estimate the multivariable association between public perceptions, personal characteristics, and the three preventive behaviors. We included the following variables in our analyses: sex, age (65 years or older), residential area, living arrangements, income level (five groups), perceived probability, perceived severity, perceived effectiveness, and feeling anxiety, and the variable of region as a covariate. All statistical analyses were performed using R version 4.0.0 (R Foundation for Statistical Computing, Vienna, Austria). The codes used for the statistical analysis are uploaded to GitHub (https://github.com/fujichaaan/covid19_opendata).

## Results

Table 2 shows the respondents’ characteristics by country. The proportions of respondents who answered that he/she wore a mask (at the time of the survey) were above 85% in three Asian countries and Italy, compared with 73.3% and 27.6% in the UK and the USA, respectively. Over 90% of respondents washed their hands/used hand sanitizer in all countries except for Japan. Finally, the proportions of respondents who avoided social gatherings ranged from 81.5% in the USA to 92.6% in Japan.

**Table 2:** Public perception and personal characteristics of participants by six countries

	China (*n* = 994)	Italy (*n* = 1020)	Japan (*n* = 981)	Korea (*n* = 918)	UK (*n* = 994)	US (*n* = 1038)
Over 65 years old (*n*)	117 (11.8%)	180 (17.6%)	196 (20.0%)	133 (14.5%)	157 (15.8%)	239 (23.0%)
Male (*n*)	487 (49.0%)	522 (51.2%)	474 (48.3%)	474 (51.6%)	489 (49.2%)	454 (43.7%)
Living urban (*n*)	723 (72.7%)	607 (59.5%)	271 (27.6%)	554 (60.3%)	339 (34.1%)	428 (41.2%)
Living alone (*n*)	418 (42.1%)	168 (16.5%)	218 (22.2%)	155 (16.9%)	233 (23.4%)	253 (24.4%)
Income group (*n*)						
First quintile	201 (20.2%)	170 (16.7%)	206 (21.0%)	198 (21.6%)	180 (18.1%)	181 (17.4%)
Second quintile	199 (20.0%)	179 (17.5%)	209 (21.3%)	163 (17.8%)	179 (18.0%)	195 (18.8%)
Third quintile	198 (19.9%)	244 (23.9%)	214 (21.8%)	199 (21.7%)	196 (19.7%)	218 (21.0%)
Fourth quintile	198 (19.9%)	262 (25.7%)	187 (19.1%)	200 (21.8%)	222 (22.3%)	245 (23.6%)
Fifth quintile	198 (19.9%)	165 (16.2%)	165 (16.8%)	158 (17.2%)	217 (21.8%)	199 (19.2%)
Feeling anxiety (*n*)	450 (45.3%)	468 (45.9%)	472 (48.1%)	437 (47.6%)	431 (43.4%)	447 (43.1%)
Use public transportation (*n*)	269 (27.1%)	96 (9.4%)	264 (26.9%)	332 (36.2%)	179 (18.0%)	147 (14.2%)
Perceived susceptibility (%)^a^	10.0 (16.6)	14.0 (22.7)	19.8 (22.6)	10.7 (17.5)	22.4 (26.2)	21.7 (28.4)
Perceived severity (%)^a^	35.3 (30.8)	31.3 (26.3)	27.8 (23.3)	28.5 (28.0)	31.9 (25.2)	40.3 (29.6)
Perceived effectiveness for wearing a mask (*n*)						
Not effective at all	22 (2.2%)	61 (6.0%)	69 (7.0%)	14 (1.5%)	197 (19.8%)	58 (5.6%)
Slightly effective	31 (3.1%)	54 (5.3%)	148 (15.1%)	28 (3.1%)	207 (20.8%)	107 (10.3%)
Moderately effective	105 (10.6%)	150 (14.7%)	248 (25.3%)	127 (13.8%)	272 (27.4%)	204 (19.7%)
Very effective	263 (26.5%)	272 (26.7%)	213 (21.7%)	394 (42.9%)	164 (16.5%)	285 (27.5%)
Extremely effective	573 (57.6%)	483 (47.4%)	303 (30.9%)	355 (38.7%)	154 (15.5%)	384 (37.0%)
Perceived effectiveness for washing hands (*n*)						
Not effective at all	19 (1.9%)	18 (1.8%)	19 (1.9%)	9 (1.0%)	21 (2.1%)	15 (1.4%)
Slightly effective	32 (3.2%)	46 (4.5%)	92 (9.4%)	30 (3.3%)	60 (6.0%)	47 (4.5%)
Moderately effective	151 (15.2%)	153 (15.0%)	178 (18.1%)	141 (15.4%)	202 (20.3%)	117 (11.3%)
Very effective	444 (44.7%)	379 (37.2%)	316 (32.2%)	479 (52.2%)	322 (32.4%)	295 (28.4%)
Extremely effective	348 (35.0%)	424 (41.6%)	376 (38.3%)	259 (28.2%)	389 (39.1%)	564 (54.3%)
Perceived effectiveness for avoiding social gathering (*n*)						
Not effective at all	34 (3.4%)	31 (3.0%)	59 (6.0%)	19 (2.1%)	34 (3.4%)	37 (3.6%)
Slightly effective	32 (3.2%)	38 (3.7%)	69 (7.0%)	35 (3.8%)	38 (3.8%)	64 (6.2%)
Moderately effective	133 (13.4%)	71 (7.0%)	163 (16.6%)	90 (9.8%)	125 (12.6%)	118 (11.4%)
Very effective	265 (26.7%)	233 (22.8%)	209 (21.3%)	339 (36.9%)	256 (25.8%)	250 (24.1%)
Extremely effective	530 (53.3%)	647 (63.4%)	481 (49.0%)	435 (47.4%)	541 (54.4%)	569 (54.8%)
Wearing a mask (*n*)						
Never	24 (2.4%)	75 (7.4%)	59 (6.0%)	27 (2.9%)	661 (66.5%)	206 (19.8%)
Rarely	48 (4.8%)	55 (5.4%)	63 (6.4%)	26 (2.8%)	59 (5.9%)	71 (6.8%)
Sometimes	68 (6.8%)	66 (6.5%)	98 (10.0%)	41 (4.5%)	90 (9.1%)	143 (13.8%)
Very often	209 (21.0%)	164 (16.1%)	184 (18.8%)	155 (16.9%)	76 (7.6%)	212 (20.4%)
Always	645 (64.9%)	660 (64.7%)	577 (58.8%)	669 (72.9%)	108 (10.9%)	406 (39.1%)
Handwashing/using hand sanitizers (*n*)						
Never	8 (0.8%)	13 (1.3%)	47 (4.8%)	28 (3.1%)	7 (0.7%)	16 (1.5%)
Rarely	56 (5.6%)	15 (1.5%)	52 (5.3%)	23 (2.5%)	19 (1.9%)	26 (2.5%)
Sometimes	60 (6.0%)	38 (3.7%)	75 (7.6%)	48 (5.2%)	75 (7.5%)	65 (6.3%)
Very often	320 (32.2%)	285 (27.9%)	199 (20.3%)	279 (30.4%)	303 (30.5%)	228 (22.0%)
Always	550 (55.3%)	669 (65.6%)	608 (62.0%)	540 (58.8%)	590 (59.4%)	703 (67.7%)
Avoiding social gathering (*n*)						
Never	695 (69.9%)	864 (84.7%)	806 (82.2%)	664 (72.3%)	742 (74.6%)	698 (67.2%)
Rarely	167 (16.8%)	58 (5.7%)	102 (10.4%)	116 (12.6%)	112 (11.3%)	148 (14.3%)
Sometimes	73 (7.3%)	42 (4.1%)	42 (4.3%)	47 (5.1%)	73 (7.3%)	87 (8.4%)
Very often	41 (4.1%)	27 (2.6%)	13 (1.3%)	37 (4.0%)	34 (3.4%)	65 (6.3%)
Always	18 (1.8%)	29 (2.8%)	18 (1.8%)	54 (5.9%)	33 (3.3%)	40 (3.9%)

^a^Values are expressed as mean and standard deviation

Table 3 shows the associations between public perceptions, personal characteristics, and wearing a mask by country. Lower odds ratios (ORs) were observed with a 10% increment of perceived susceptibility in China (OR, 0.83; 95% confidence interval [CI], 0.74–0.94) and Korea (OR, 0.86; 95%CI, 0.75–1.00), and the UK (OR, 1.08; 95 %Cl, 1.01–1.14). High perceived severity was associated with wearing a mask in the USA (OR, 1.07; 95%CI, 1.02–1.14), whereas a negative association was observed in China (OR, 0.91; 95 %Cl, 0.83–0.99). Except for China, those who perceived that wearing a mask was extremely effective had higher ORs in Italy (OR, 4.14; 95%CI, 2.08–8.02), Japan (OR, 3.59; 95%CI, 1.75–7.30), Korea (OR, 7.89; 95%CI, 1.91–31.63), the UK (OR, 9.23; 95%CI, 5.14–17.31), and the USA (OR, 4.81; 95%CI, 2.61–8.92). Older people were less likely to wear a mask in the UK (OR, 0.29; 95%CI, 0.15–0.51). Male participants were less likely to wear a mask only in Japan (OR, 0.30; 95%CI, 0.19–0.46). Those who lived in urban areas had high ORs in Japan (OR, 1.70; 95%CI, 1.03–2.90), the UK (OR, 1.52; 95%CI, 1.09–2.11), and the USA (OR, 1.59; 95%CI, 1.15–2.20). The participants in the fifth income quintile were more likely to wear a mask in Korea (OR, 9.19; 95%CI, 2.50–59.50), and the USA (OR, 3.24; 95%CI, 1.93–5.53). Feeling anxiety was positively associated with wearing a mask in Japan (OR, 2.11; 95%CI, 1.37–3.31), whereas those who were living alone were less likely to wear a mask (OR, 0.43; 95%CI, 0.28–0.68).

**Table 3:** Associations between public perceptions, personal characteristics, and wearing a mask

	China	Italy	Japan	Korea	UK	US
	OR (95%CI)	OR (95%CI)	OR (95%CI)	OR (95%CI)	OR (95%CI)	OR (95%CI)
Age group						
Under 65 years old	1.00 (Ref.)	1.00 (Ref.)	1.00 (Ref.)	1.00 (Ref.)	1.00 (Ref.)	1.00 (Ref.)
Over 65 years old	1.05 (0.49–2.45)	0.92 (0.54–1.61)	1.41 (0.83–2.46)	0.85 (0.39–2.07)	0.29 (0.15–0.51)^b^	1.29 (0.89–1.88)
Sex						
Female	1.00 (Ref.)	1.00 (Ref.)	1.00 (Ref.)	1.00 (Ref.)	1.00 (Ref.)	1.00 (Ref.)
Male	0.71 (0.41–1.22)	0.90 (0.60–1.33)	0.30 (0.19–0.46)^b^	0.57 (0.29–1.06)	1.29 (0.93–1.79)	1.02 (0.74–1.40)
Use public transportation						
No	1.00 (Ref.)	1.00 (Ref.)	1.00 (Ref.)	1.00 (Ref.)	1.00 (Ref.)	1.00 (Ref.)
Yes	0.19 (0.11–0.31)^b^	0.32 (0.19–0.56)^b^	1.31 (0.81–2.16)	1.80 (0.94–3.65)	2.12 (1.45–3.10)^b^	0.89 (0.56–1.42)
Living urban						
No	1.00 (Ref.)	1.00 (Ref.)	1.00 (Ref.)	1.00 (Ref.)	1.00 (Ref.)	1.00 (Ref.)
Yes	0.87 (0.45–1.61)	1.37 (0.92–2.03)	1.70 (1.03–2.90)^d^	0.68 (0.35–1.27)	1.52 (1.09–2.11)^d^	1.59 (1.15–2.20)^c^
Living alone						
No	1.00 (Ref.)	1.00 (Ref.)	1.00 (Ref.)	1.00 (Ref.)	1.00 (Ref.)	1.00 (Ref.)
Yes	0.77 (0.45–1.34)	0.83 (0.51–1.38)	0.43 (0.28–0.68)^b^	1.18 (0.55–2.74)	0.88 (0.59–1.30)	1.33 (0.93–1.90)
Income group						
1st quintile	1.00 (Ref.)	1.00 (Ref.)	1.00 (Ref.)	1.00 (Ref.)	1.00 (Ref.)	1.00 (Ref.)
2nd quintile	1.11 (0.52–2.38)	1.31 (0.69–2.52)	1.00 (0.53–1.87)	2.21 (0.9–5.99)	1.17 (0.69–1.98)	1.6 (1.02–2.52)^d^
3rd quintile	1.37 (0.61–3.16)	1.11 (0.61–2.02)	0.69 (0.38–1.24)	3.03 (1.26–8.13)^d^	1.24 (0.75–2.09)	2.27 (1.44–3.62)^b^
4th quintile	1.27 (0.57–2.90)	1.42 (0.79–2.56)	1.74 (0.84–3.72)	1.85 (0.81–4.38)	1.06 (0.63–1.80)	2.05 (1.31–3.22)^c^
5th quintile	2.34 (0.94–6.30)	1.48 (0.76–2.93)	1.06 (0.51–2.24)	9.19 (2.50–59.50)^c^	1.02 (0.61–1.71)	3.24 (1.93–5.53)^b^
Feeling anxiety						
No	1.00 (Ref.)	1.00 (Ref.)	1.00 (Ref.)	1.00 (Ref.)	1.00 (Ref.)	1.00 (Ref.)
Yes	1.40 (0.81–2.46)	0.9 (0.60–1.34)	2.11 (1.37–3.31)^b^	1.67 (0.89–3.24)	1.04 (0.76–1.43)	1.31 (0.96–1.80)
Perceived susceptibility^a^	0.83 (0.74–0.94)^c^	1.07 (0.97–1.19)	0.94 (0.86–1.03)	0.86 (0.75–1.00)^d^	1.08 (1.01–1.14)^d^	1.00 (0.94–1.07)
Perceived severity^a^	0.91 (0.83–0.99)^d^	1.05 (0.97–1.13)	1.00 (0.92–1.10)	0.97 (0.87–1.10)	1.05 (0.99–1.12)	1.07 (1.02–1.14)^d^
Perceived effectiveness						
Not effective at all	1.00 (Ref.)	1.00 (Ref.)	1.00 (Ref.)	1.00 (Ref.)	1.00 (Ref.)	1.00 (Ref.)
Slightly effective	1.11 (0.04–32.35)	1.51 (0.63–3.74)	2.16 (1.01–4.62)^d^	2.66 (0.52–14.81)	1.92 (1.03–3.68)^d^	1.66 (0.85–3.27)
Moderately effective	0.23 (0.01–1.51)	1.09 (0.54–2.17)	2.59 (1.28–5.19)^c^	2.65 (0.65–10.52)	4.08 (2.35–7.40)^b^	2.45 (1.32–4.58)^c^
Very effective	0.50 (0.02–3.16)	3.79 (1.83–7.72)^b^	3.64 (1.69–7.87)^b^	7.99 (1.96–31.42)^c^	6.03 (3.35–11.31)^b^	3.03 (1.65–5.56)^b^
Extremely effective	0.51 (0.03–3.14)	4.14 (2.08–8.02)^b^	3.59 (1.75–7.30)^b^	7.89 (1.91–31.63)^c^	9.23 (5.14–17.31)^b^	4.81 (2.61–8.92)^b^

*COVID-19*, coronavirus diesease 2019; *OR*, odds ratio; *95%CI*, 95% confidence intervals

^a^The odds ratios indicate indicate a propability of mask wearing with a 10% increase in each perception (%)

^b^*P*-value < 0.001

^c^*P*-value < 0.01

^d^*P*-value < 0.05

Table 4 shows the associations between public perceptions, personal characteristics, and handwashing/using hand sanitizers by country. Inverse associations were observed between hand sanitizing and perceived susceptibility in China (OR, 0.81; 95%CI, 0.71–0.91) and Korea (OR, 0.77; 95%CI, 0.66–0.90). Those who had higher perceived effectiveness were more likely to wash their hands in Italy (OR, 16.39; 95%CI, 3.56–70.18), Japan (OR, 12.24; 95%CI, 4.03–37.35), Korea (OR, 12.41; 95%CI, 2.02–76.39), the UK (OR, 18.04; 95%CI, 2.60–152.78), and the USA (OR, 10.56; 95%CI, 2.21–44.32). In line with wearing a mask, older people were more likely to wash their hands in Japan (OR, 2.47; 95%CI, 1.27–5.21). Male respondents were less likely to wash their hands in Japan (OR, 0.32; 95%CI, 0.19–0.53) and China (OR, 0.56; 95%CI, 0.31–0.98). Those who lived alone were less likely to wash their hands in Japan (OR, 0.50; 95%CI, 0.31–0.82). Feeling anxiety was positively associated with washing hands in China (OR, 2.05; 95%CI, 1.15–3.80) and Japan (OR, 1.74; 95%CI, 1.09–2.83). In Korea only, the participants in the fifth income quintile were more likely to wash hands (OR, 3.50; 95%CI, 1.27–11.37)

**Table 4:** Associations between public perceptions, personal characteristics, and handwashing/using hand sanitizers

	China	Italy	Japan	Korea	UK	US
	OR (95%CI)	OR (95%CI)	OR (95%CI)	OR (95%CI)	OR (95%CI)	OR (95%CI)
Age group						
Under 65 years old	1.00 (Ref.)	1.00 (Ref.)	1.00 (Ref.)	1.00 (Ref.)	1.00 (Ref.)	1.00 (Ref.)
Over 65 years old	1.75 (0.71–5.11)	1.74 (0.56–7.64)	2.47 (1.27–5.21)^d^	0.55 (0.26–1.28)	3.50 (0.66–64.78)	2.20 (0.70–9.77)
Sex						
Female	1.00 (Ref.)	1.00 (Ref.)	1.00 (Ref.)	1.00 (Ref.)	1.00 (Ref.)	1.00 (Ref.)
Male	0.56 (0.31–0.98)^d^	0.55 (0.23–1.23)	0.32 (0.19–0.53)^b^	0.52 (0.26–1.00)	0.43 (0.16–1.06)	0.47 (0.21–1.01)
Use public transportation						
No	1.00 (Ref.)	1.00 (Ref.)	1.00 (Ref.)	1.00 (Ref.)	1.00 (Ref.)	1.00 (Ref.)
Yes	0.78 (0.44–1.43)	0.89 (0.30–3.34)	1.62 (0.96–2.82)	1.33 (0.70–2.63)	0.53 (0.22–1.34)	0.51 (0.24–1.10)
Living urban						
No	1.00 (Ref.)	1.00 (Ref.)	1.00 (Ref.)	1.00 (Ref.)	1.00 (Ref.)	1.00 (Ref.)
Yes	1.29 (0.66–2.43)	1.31 (0.57–2.96)	0.66 (0.40–1.10)	0.51 (0.25–1.00)	0.57 (0.23–1.39)	1.01 (0.48–2.09)
Living alone						
No	1.00 (Ref.)	1.00 (Ref.)	1.00 (Ref.)	1.00 (Ref.)	1.00 (Ref.)	1.00 (Ref.)
Yes	0.73 (0.41–1.32)	0.52 (0.22–1.36)	0.50 (0.31–0.82)^c^	1.10 (0.49–2.65)	2.49 (0.91–8.15)	0.86 (0.41–1.90)
Income group						
1st quintile	1.00 (Ref.)	1.00 (Ref.)	1.00 (Ref.)	1.00 (Ref.)	1.00 (Ref.)	1.00 (Ref.)
2nd quintile	0.51 (0.21–1.15)	0.52 (0.14–1.81)	1.02 (0.53–1.96)	2.71 (1.00–8.69)	1.00 (0.27–3.61)	2.35 (0.74–8.33)
3rd quintile	0.96 (0.37–2.45)	0.96 (0.26–3.42)	1.49 (0.75–2.99)	2.25 (0.93–5.86)	1.87 (0.46–8.05)	4.33 (1.17–21.04)^d^
4th quintile	0.73 (0.28–1.89)	1.10 (0.29–3.93)	2.57 (1.15–6.11)^d^	2.00 (0.86–4.86)	4.64 (0.90–35.01)	1.04 (0.38–2.70)
5th quintile	0.90 (0.33–2.51)	0.88 (0.21–3.90)	1.11 (0.52–2.38)	3.50 (1.27–11.37)^d^	1.09 (0.31–3.53)	1.63 (0.55–4.88)
Feeling anxiety						
No	1.00 (Ref.)	1.00 (Ref.)	1.00 (Ref.)	1.00 (Ref.)	1.00 (Ref.)	1.00 (Ref.)
Yes	2.05 (1.15–3.80)^d^	1.36 (0.59–3.35)	1.74 (1.09–2.83)^d^	1.16 (0.61–2.22)	0.84 (0.35–2.07)	1.06 (0.52–2.23)
Perceived susceptibility^a^	0.81 (0.71–0.91)^b^	0.99 (0.84–1.18)	0.98 (0.90–1.09)	0.77 (0.66–0.90)^b^	1.05 (0.89–1.27)	0.91 (0.80–1.03)
Perceived severity^a^	1.02 (0.93–1.13)	1.05 (0.90–1.25)	0.96 (0.88–1.06)	1.12 (0.98–1.30)	1.01 (0.85–1.21)	0.92 (0.81–1.06)
Perceived effectiveness						
Not effective at all	1.00 (Ref.)	1.00 (Ref.)	1.00 (Ref.)	1.00 (Ref.)	1.00 (Ref.)	1.00 (Ref.)
Slightly effective	0.40 (0.02–2.92)	1.85 (0.41–7.86)	3.00 (0.97–9.33)	4.55 (0.65–35.65)	1.01 (0.18–4.64)	1.45 (0.28–6.80)
Moderately effective	0.38 (0.02–2.08)	6.84 (1.51–28.73)^c^	4.53 (1.53–13.43)^c^	2.10 (0.40–10.27)	3.37 (0.62–15.17)	4.26 (0.82–20.61)
Very effective	0.79 (0.04–4.26)	16.15 (3.52–68.93)^b^	7.75 (2.60–23.09)^b^	8.28 (1.57–41.10)^c^	3.35 (0.64–13.93)	6.35 (1.32–26.81)^d^
Extremely effective	3.68 (0.18–25.36)	16.39 (3.56–70.18)^b^	12.24 (4.03–37.35)^b^	12.41 (2.02–76.39)^c^	18.04 (2.60–152.78)^c^	10.56 (2.21–44.32)^c^

*COVID-19*, coronavirus diesease 2019; *OR*, odds ratio; *95%CI,* 95% confidence intervals

^a^The odds ratios indicate indicate a propability of handwashing with a 10% increase in each perception (%)

^b^*P*-value < 0.001

^c^*P*-value < 0.01

^d^*P*-value < 0.05

Table 5 shows the associations between public perceptions, personal characteristics, and avoiding social gatherings in the six different countries. Perceived susceptibility was inversely associated with avoiding social gatherings in China (OR, 0.74; 95%CI, 0.66–0.83), Japan (OR, 0.85; 95%CI, 0.77–0.95), Korea (OR, 0.81; 95%CI, 0.73–0.91), and the USA (OR, 0.83; 95%CI, 0.77–0.90). Participants with high perceived severity were more likely to avoid social gatherings in China (OR, 1.27; 95%CI, 1.16–1.41) and the USA (OR, 1.11; 95%CI, 1.03–1.19). Those who had higher perceived effectiveness were more likely to avoid social gatherings in China (OR, 3.79; 95%CI, 1.28–10.23), Korea (OR, 6.18; 95%CI, 1.77–20.60), the UK (OR, 4.45; 95%CI, 1.63–11.63), and the USA (OR, 4.34; 95%CI, 1.84–9.95), but neither in Italy nor in Japan. Older people tended to avoid social gatherings in China (OR, 2.78; 95%CI, 1.29–6.55). Male participants were less likely to avoid social gatherings in Japan (OR, 0.33; 95%CI, 0.19–0.58) and the USA (OR, 0.54; 95%CI, 0.36–0.82). Although those who lived in urban areas were more likely to avoid social gatherings in Korea (OR, 2.24; 95%CI, 1.45–3.46), an inverse association was found in Italy (OR, 0.43; 95%CI, 0.24–0.75) and the UK (OR, 0.49; 95%CI, 0.31–0.78). Participants with higher incomes were less likely to avoid social gatherings in the USA (OR, 0.49; 95%CI, 0.24–0.97).

**Table 5:** Associations between public perceptions, personal characteristics, and avoiding social gathering

	China	Italy	Japan	Korea	UK	US
	OR (95%CI)	OR (95%CI)	OR (95%CI)	OR (95%CI)	OR (95%CI)	OR (95%CI)
Age group						
Under 65 years old	1.00 (Ref.)	1.00 (Ref.)	1.00 (Ref.)	1.00 (Ref.)	1.00 (Ref.)	1.00 (Ref.)
Over 65 years old	2.78 (1.29–6.55)^d^	1.89 (0.84–4.88)	1.15 (0.57–2.49)	1.22 (0.65–2.41)	1.28 (0.60–3.01)	1.38 (0.80–2.47)
Sex						
Female	1.00 (Ref.)	1.00 (Ref.)	1.00 (Ref.)	1.00 (Ref.)	1.00 (Ref.)	1.00 (Ref.)
Male	0.81 (0.52–1.26)	0.82 (0.49–1.37)	0.33 (0.19–0.58)^b^	0.93 (0.60–1.42)	0.91 (0.57–1.45)	0.54 (0.36–0.82)^c^
Use public transportation						
No	1.00 (Ref.)	1.00 (Ref.)	1.00 (Ref.)	1.00 (Ref.)	1.00 (Ref.)	1.00 (Ref.)
Yes	0.09 (0.06–0.15)^b^	0.07 (0.04–0.12)^b^	0.23 (0.13–0.39)^b^	0.15 (0.10–0.23)^b^	0.07 (0.04–0.11)^b^	0.07 (0.04–0.11)^b^
Living urban						
No	1.00 (Ref.)	1.00 (Ref.)	1.00 (Ref.)	1.00 (Ref.)	1.00 (Ref.)	1.00 (Ref.)
Yes	1.64 (0.99–2.70)	0.43 (0.24–0.75)^c^	0.88 (0.51–1.55)	2.24 (1.45–3.46)^b^	0.49 (0.31–0.78)^c^	1.45 (0.96–2.24)
Living alone						
No	1.00 (Ref.)	1.00 (Ref.)	1.00 (Ref.)	1.00 (Ref.)	1.00 (Ref.)	1.00 (Ref.)
Yes	0.84 (0.53–1.34)	0.51 (0.29–0.94)^d^	1.12 (0.61–2.16)	1.10 (0.62–2.04)	1.75 (1.01–3.11)	0.82 (0.51–1.32)
Income group						
1st quintile	1.00 (Ref.)	1.00 (Ref.)	1.00 (Ref.)	1.00 (Ref.)	1.00 (Ref.)	1.00 (Ref.)
2nd quintile	0.95 (0.48–1.89)	0.64 (0.27–1.50)	2.02 (0.81–5.06)	0.90 (0.46–1.76)	0.75 (0.35–1.59)	0.68 (0.34–1.36)
3rd quintile	0.53 (0.27–1.05)	0.71 (0.31–1.58)	1.12 (0.48–2.56)	0.65 (0.35–1.19)	0.76 (0.36–1.58)	0.81 (0.41–1.61)
4th quintile	0.96 (0.46–2.01)	0.75 (0.33–1.67)	0.94 (0.40–2.14)	1.08 (0.55–2.14)	0.81 (0.37–1.77)	0.64 (0.33–1.23)
5th quintile	1.10 (0.52–2.36)	0.95 (0.38–2.43)	1.15 (0.45–2.93)	0.80 (0.40–1.60)	0.73 (0.34–1.51)	0.49 (0.24–0.97)^d^
Feeling anxiety						
No	1.00 (Ref.)	1.00 (Ref.)	1.00 (Ref.)	1.00 (Ref.)	1.00 (Ref.)	1.00 (Ref.)
Yes	1.10 (0.70–1.73)	0.63 (0.37–1.06)	1.10 (0.64–1.87)	1.10 (0.73–1.67)	1.19 (0.75–1.91)	1.12 (0.75–1.70)
Perceived susceptibility^a^	0.74 (0.66–0.83)^b^	0.96 (0.86–1.07)	0.85 (0.77–0.95)^c^	0.81 (0.73–0.91)^b^	0.97 (0.89–1.06)	0.83 (0.77–0.90)^b^
Perceived severity^a^	1.27 (1.16–1.41)^b^	0.98 (0.89–1.09)	0.97 (0.87–1.08)	1.05 (0.97–1.14)	0.92 (0.84–1.00)	1.11 (1.03–1.19)^c^
Perceived effectiveness						
Not effective at all	1.00 (Ref.)	1.00 (Ref.)	1.00 (Ref.)	1.00 (Ref.)	1.00 (Ref.)	1.00 (Ref.)
Slightly effective	0.73 (0.20–2.66)	0.70 (0.17–2.68)	0.42 (0.10–1.53)	1.39 (0.33–5.64)	1.46 (0.42–5.05)	2.25 (0.81–6.27)
Moderately effective	1.09 (0.36–3.02)	0.95 (0.26–3.25)	0.41 (0.11–1.21)	2.53 (0.68–9.10)	0.85 (0.30–2.27)	3.86 (1.48–9.97)^c^
Very effective	2.39 (0.79–6.56)	4.48 (1.25–14.99)^d^	0.86 (0.22–2.74)	3.38 (0.97–11.21)^d^	2.08 (0.76–5.52)	4.16 (1.70–10.00)^c^
Extremely effective	3.79 (1.28–10.23)^d^	3.09 (0.94–9.09)	1.28 (0.34–3.82)	6.18 (1.77–20.60)^c^	4.45 (1.63–11.63)^c^	4.34 (1.84–9.95)^b^

*COVID-19*, coronavirus diesease 2019; *OR*, odds ratio; *95%CI*, 95% confidence intervals

^a^The odds ratios indicate indicate a propability of avoiding gathering with a 10% increase in each perception (%)

^b^*P*-value < 0.001

^c^*P*-value < 0.01

^d^*P*-value < 0.05

Table 6 shows the associations between behavioral changes and the three preventive measures. Those who changed their behaviors because of recommendations from doctors were more likely to wear a mask in China (OR, 2.04; 95%CI, 1.12–3.78), Italy (OR, 2.02; 95%CI, 1.34–3.06), Japan (OR, 2.84; 95%CI, 1.34–7.01), and the USA (OR, 1.82; 95%CI, 1.33–2.50). The same trend was observed with handwashing/using hand sanitizers in China (OR, 1.70; 95%CI, 0.92–3.20), Italy (OR, 2.42; 95%CI, 0.98–6.34), and Korea (OR, 2.85; 95%CI, 0.95–12.42). Regarding avoiding social gatherings, a significant association with recommendations from doctors/public health officials the USA (OR, 2.14; 95%CI, 1.40–3.28). Statistically significant associations were observed only between behavioral changes triggered by politicians and wearing masks in the USA (OR, 1.63; 95%CI, 1.15–2.33) and avoiding social gatherings in the UK (OR, 1.70; 95%CI, 1.05–2.79). Japan was the only country in which a statistically significant positive association was observed between behavioral changes triggered by conformity (OR, 5.18; 95%CI, 1.54–32.35) and recommendations by family members to wear a mask (OR, 3.42; 95%CI, 1.19–14.48). On the other hand, in the UK, wearing a mask were inversely associated with recommendations from doctors (OR, 0.66; 95%CI, 0.48–0.90) or politicians (OR, 0.56; 95%CI, 0.41–0.77).

**Table 6:** Associations between conformity and recommendations from others and three preventive behaviors

	China	Italy	Japan	Korea	UK	US
	OR (95%CI)	OR (95%CI)	OR (95%CI)	OR (95%CI)	OR (95%CI)	OR (95%CI)
Wearing a mask						
Conformity	2.36 (0.74–10.74)	1.13 (0.45–3.49)	5.18 (1.54–32.35)^c^	2.62 (0.83–12.08)	0.78 (0.49–1.21)	1.29 (0.83–2.05)
Recommendations from family/friends	1.33 (0.70–2.70)	0.68 (0.33–1.53)	3.42 (1.19–14.48)^c^	1.05 (0.42–3.10)	1.41 (0.90–2.19)	1.27 (0.88–1.84)
Recommendations from doctors/public health officials	2.04 (1.12–3.78)^c^	2.02 (1.34–3.06)^a^	2.84 (1.34–7.01)^c^	1.24 (0.51–3.53)	0.66 (0.48–0.90)^b^	1.82 (1.33–2.50)^a^
Recommendations from politicians	1.00 (0.47–2.31)	1.60 (0.98–2.72)	1.39 (0.67–3.30)	1.09 (0.26–8.33)	0.56 (0.41–0.77)^a^	1.63 (1.15–2.33)^b^
Handwashing/using hand sanitizers						
Conformity	0.59 (0.23–1.73)	0.94 (0.17–17.83)	1.22 (0.52–3.39)	1.43 (0.51–5.22)	0.44 (0.18–1.21)	0.70 (0.30–1.78)
Recommendations from family/friends	0.96 (0.51–1.89)	0.60 (0.15–3.99)	1.52 (0.62–4.61)	1.71 (0.60–6.32)	1.44 (0.39–9.40)	0.90 (0.41–2.15)
Recommendations from doctors/public health officials	1.70 (0.92–3.20)	2.42 (0.98–6.34)	1.75 (0.85–4.13)	2.85 (0.95–12.42)	0.97 (0.40–2.37)	1.71 (0.81–3.79)
Recommendations from politicians	1.74 (0.74–4.88)	0.60 (0.24–1.63)	1.21 (0.55–3.05)	1.83 (0.32–36.37)	0.91 (0.38–2.24)	2.68 (0.99–9.42)
Avoiding social gathering						
Conformity	0.78 (0.34–1.93)	0.76 (0.26–2.67)	1.41 (0.54–1.51)	1.11 (0.58–2.27)	0.85 (0.45–1.66)	1.20 (0.70–2.15)
Recommendations from family/friends	1.17 (0.68–2.05)	0.48 (0.21–1.24)	1.13 (0.46–3.25)	0.73 (0.38–1.46)	0.72 (0.38–1.41)	1.23 (0.76–2.04)
Recommendations from doctors/public health officials	1.22 (0.75–1.98)	1.54 (0.90–2.64)	1.44 (0.64–3.70)	1.40 (0.77–2.68)	1.05 (0.66–1.68)	2.14 (1.40–3.28)^a^
Recommendations from politicians	0.66 (0.36–1.22)	1.16 (0.62–2.28)	1.09 (0.46–2.93)	0.94 (0.33–3.46)	1.70 (1.05–2.79)^b^	1.44 (0.90–2.34)

Adjusted for gender, age (65 or more), residential area, living arrangement, income levels (five groups), perceived probability, perceived severity, perceived effectiveness, feeling anxiety, and region

*COVID-19*, coronavirus diesease 2019; *OR*, odds ratio; *95%CI*, 95% confidence intervals

^a^*P*-value < 0.001

^b^*P*-value < 0.01

^c^*P*-value < 0.05

## Discussion

In this study, we examined the association between perceptions, personal characteristics, recommendations from others, and three preventive behaviors in six countries during the COVID-19 pandemic. According to the HBM, a canonical model of behavioral medicine, the three individual perceptions examined here affect the preventive measures to be taken [20]. Among these three perceptions, we found that perceived effectiveness was a common driving factor for engaging in preventive behaviors in all six countries. Regarding the other two perceptions, we found that the effects varied across countries. Associations between other individual demographic and socioeconomic traits and preventive behaviors were also heterogeneous across countries.

In behavioral medicine, it is widely accepted that perceived susceptibility (likelihood of contracting a disease or developing a condition), perceived severity (seriousness of an illness), and perceived effectiveness (effectiveness of a suggested preventive measure) are key components in taking preventive measures and thereby controlling infectious disease outbreaks. During the influenza A virus subtype H1N1 pandemic of 2009, both perceived susceptibility and severity were found to be significant factors in persuading the public to change their preventive behavior [10–12]. Contrary to our theoretical expectations, however, our results showed inverse associations between perceived susceptibility and severity and preventive behaviors in some countries. This could potentially be attributed to reverse causality, i.e., we hypothesized that because of the cross-sectional design, the respondents who took preventive behaviors were more likely to perceive a low likelihood of having been infected. In addition, the design of the questionnaire might have failed to gauge empirically the concept of perceived susceptibility. Therefore, the association observed in this study between preventive behaviors and perceived susceptibility requires careful interpretation. Through the lens of the HBM, perceived effectiveness is a concept similar to the construct of “perceived benefit.” Previous studies have reported positive associations between an individual’s perceived effectiveness and preventive behaviors, e.g., during the SARS epidemic of 2003, people who perceived the effectiveness of preventive measures in Hong Kong were more likely to wear a mask (OR: 7.15, 95%CI: 4.25–12.05), wash their hands (OR: 32.00, 95%CI: 13.88–73.78), and avoid crowded places (OR: 31.56, 95%CI: 15.61–63.82) [14]. Considering the observed homogeneous effects of perceived effectiveness across preventive behaviors and countries, government recommendations and social communications should enhance their effectiveness to promote better compliance.

Although the results of this study were not uniform across all countries, personal characteristics, including sex, age, and income groups, were associated with all three preventive behaviors. Similar to the previously reported effect of sex on health behaviors, female participants tended to engage in more preventive behaviors than did males. Consistent with previous studies on SARS [10, 11, 14, 21], we also found that older adults were more likely to take preventive measures. High-income groups were associated with an increased probability of taking preventive behaviors, but educational experience and ethnicity may have been potential confounders underlying this association [22–24]. A study on sociodemographic factors in response to SARS in New York suggested that high-income groups were more likely to access accurate information [25]. Therefore, high-income individuals may take more appropriate actions when faced with emerging diseases. We also assessed the effect of living environment on taking preventive behaviors. Given the high passenger density and difficulty of social distancing in public transportation, we would expect that those who use public transportation are more likely to take preventive measures. However, our results showed that the use of public transportation (as of the survey date) was negatively associated with the probability to wear a mask. This unexpected result may be explained by the downward bias of the coefficient estimate driven by the potential negative correlation between the covariate and unobserved variables. For instance, an individual who is more seriously concerned about a disease and thus more inclined to wear a mask may be more likely to avoid using public transportation. To verify this potential endogeneity problem, we also ran a regression involving the use of public transportation before the pandemic, which may be correlated with the use of transportation after the pandemic will be independent of the unobserved variables. The results yielded theoretically consistent estimates, i.e., the use of public transportation was associated with a higher probability of wearing a mask. In addition, feeling anxiety may have been associated with preventive behaviors in some countries, which was consistent with the results observed in previous studies [21, 25].

Behavioral changes triggered by conformity and recommendations from others showed different impacts on preventive behaviors across countries, and the impacts also varied across all three preventive behaviors within each country. However, the bottom line is that individuals who changed their behavior because of recommendations from doctors/public health officials were more likely to take preventive behaviors in many countries, e.g., wearing a mask in China, Italy, Japan, and the USA, and avoiding social gatherings in the USA. On the other hand, recommendations by politicians did not significantly affect preventive behaviors, except for wearing a mask in the USA and avoiding social gatherings in the UK. In many countries during the pandemic, local governments have been holding regular press conferences to provide daily updates on the pandemic and call for preventive measures. Our results suggest that the engagement of medical professionals in addressing the significance of preventive measures is more effective. Another interesting finding was that individuals in Japan who changed their behavior after taking conformity into account were more likely to wear a mask. This may reflect cultural norms in Japan, in which individual behavior is affected greatly by the behaviors of others around him/her [26, 27].

Several methodological issues need to be mentioned as limitations. First, this study was conducted in a limited sample population, which may affect the external and internal validity. Ideally, an epidemiological study should be conducted on randomly selected participants from the general population. This online survey was designed to retain national representation regarding basic demographic variables, which would alleviate a fundamental sampling bias. However, detailed information on response rate, recruiting method, and the amount of remuneration were not available, which constitute potential sources of bias. Despite this, we consider that this sampling method should be accepted given the nature of an emergency survey. Second, important covariates such as ethnicity, educational experience, and preexisting comorbidities were not available in this study. Previous studies have reported that ethnicity is a major factor in preventive behaviors [23]. Third, the design of questionnaire used in this study may be crucial for interpreting the results. In particular, the question item on perceived susceptibility to COVID-19 was likely critically problematic. In this survey, participants were asked “What do you think is the probability that you are or have been infected with COVID-19?,” i.e., the current susceptibility for COVID-19. However, in general, a question on perceived susceptibility should ask about the possibility of being infected in the future. In addition, the questionnaire had only one question regarding personal perceptions, whereas conventional methods usually employ multiple questions to assess individual perceptions more precisely. Due to the poorly defined questionnaire and the cross-sectional design of this study, some caution is needed in interpreting the results, as described above. Further cross-country studies using a longitudinal design and well-structured questionnaires could be expected to overcome these limitations and provide a more accurate assessment of the structural relationships between different factors and preventive behaviors. Forth, we need to be more careful when interpreting the results, which showed wide confidence intervals. In particular, since almost all people practiced handwashing in each country, the confidence intervals for the estimates were wider than the other two preventive behaviors. We may potentially reduce the number of variables in the regressions. However, given the emphasis on comparing the results across different preventive practices, we used the same set of variables for each preventive behavior rather than making variable selection. Therefore, for a wide confidence interval, the estimates should be interpreted by integrating them with other studies. Fifth, in comparing the results among six different counties, we would need to take into account the different epidemic phases of COVID-19 in each country at the time of the survey. In the fully adjusted models, we included regional fixed effects to control for the heterogeneous infectious status within a country. However, given that the population are firstly stratified by country, it was not straightforward to incorporate the across-country heterogeneity. It is important to keep this issue in mind when interpreting the data. The infection status at the time of the online survey has been described in previous studies [19].

## Conclusions

In conclusion, first, our results suggest that, when encouraging the general public to engage in preventive measures during a pandemic, it would be effective to publicize the effectiveness of such measures. Second, associations between individual characteristics (both demographic and socioeconomic) and preventive behaviors vary across countries and preventive measures, which highlights the importance of targeting subgroups of people when preventive measures are implemented by a health administration. Third, our results suggest that incorporating different associations between individual characteristics and preventive behaviors across countries may provide more precise simulation results in mathematical modeling.

## Supplementary Information

**Additional file 1.** The online self-administered questionnaire for this six-country survey. This questionnaire was used to assess individual perceptions of COVID-19, and their behavioral change.

## Data Availability

The data are publicly available at https://osf.io/aubkc/. The codes used for the statistical analysis are uploaded to GitHub (https://github.com/fujichaaan/covid19_opendata).

## Abbreviations

CI: Confidence interval
COVID-19: Coronavirus disease 2019
HBM: Health Belief Model
OR: Odds ratio
SARS: Severe acute respiratory syndrome
SARS-CoV-2: Severe acute respiratory syndrome-coronavirus-2

## References

[1] World Health Organization. Coronavirus disease 2019 (COVID-19) situation report - 207. Accessed at https://www.who.int/docs/default-source/coronaviruse/situation-reports/20200814-covid-19-sitrep-207.pdf?sfvrsn=2f2154e6_2 on 14 August 2020.

[2] Worldometer. COVID-19 Coronavirus outbreak. Accessed at https://www.worldometers.info/coronavirus/ on 14 January 2021.

[3] CDC COVID-19 Response Team (2020). Severe outcomes among patients with coronavirus disease 2019 (COVID-19) - United States, February 12-March 16, 2020. MMWR Morb Mortal Wkly Rep.

[4] CDC COVID-19 Response Team (2020). Preliminary estimates of the prevalence of selected underlying health conditions among patients with coronavirus disease 2019 - United States, February 12-March 28, 2020. MMWR Morb Mortal Wkly Rep.

[5] Pan A, Liu L, Wang C, Guo H, Hao X, Wang Q (2020). Association of Public health interventions with the epidemiology of the COVID-19 outbreak in Wuhan, China. JAMA.

[6] Davies NG, Kucharski AJ, Eggo RM, Gimma A, Edmunds WJ (2020). Centre for the Mathematical Modelling of Infectious Diseases COVID-19 working group. Effects of non-pharmaceutical interventions on COVID-19 cases, deaths, and demand for hospital services in the UK: a modelling study. Lancet Public Health..

[7] Chu DK, Akl EA, Duda S, Solo K, Yaacoub S, Schünemann HJ (2020). COVID-19 Systematic Urgent Review Group Effort (SURGE) study authors. COVID-19 Systematic Urgent Review Group Effort (SURGE) study authors. Physical distancing, face masks, and eye protection to prevent person-to-person transmission of SARS-CoV-2 and COVID-19: a systematic review and meta-analysis. Lancet..

[8] Cowling BJ, Ali ST, Ng TWY, Tsang TK, Li JCM, Fong MW (2020). Impact assessment of non-pharmaceutical interventions against coronavirus disease 2019 and influenza in Hong Kong: an observational study. Lancet Public Health..

[9] Feng S, Shen C, Xia N, Song W, Fan M, Cowling BJ (2020). Rational use of face masks in the COVID-19 pandemic. Lancet Respir Med..

[10] Tang CS, Wong CY (2004). Factors influencing the wearing of facemasks to prevent the severe acute respiratory syndrome among Chinese in Hong Kong. Prev Med..

[11] Tang CS, Wong CY (2003). An outbreak of the severe acute respiratory syndrome: predictors of health behaviors and effect of community prevention measures in Hong Kong, China. Am J Public Health.

[12] Leung GM, Quah S, Ho LM, Ho SY, Hedley AJ, Lee HP (2004). A tale of two cities: community psychobehavioral surveillance and related impact on outbreak control in Hong Kong and Singapore during the severe acute respiratory syndrome epidemic. Infect Control Hosp Epidemiol.

[13] Bruine de Bruin W, Bennett D (2020). Relationships between initial COVID-19 risk perceptions and protective health behaviors: a national survey. Am J Prev Med.

[14] Lau JTF, Yang X, Tsui H, Kim JH (2003). Monitoring community responses to the SARS epidemic in Hong Kong: from day 10 to day 62. J Epidemiol Community Health.

[15] Pampel FC, Krueger PM, Denney JT (2010). Socioeconomic disparities in health behaviors. Annu Rev Sociol.

[16] Bertozzi AL, Franco E, Mohler G, Short MB, Sledge D. The challenges of modeling and forecasting the spread of COVID-19. Proc Natl Acad Sci USA. 2020;117:16732–8. 10.1073/pnas.2006520117 PMC7382213 32616574

[17] Kissler SM, Tedijanto C, Goldstein E, Grad YH, Lipsitch M (2020). Projecting the transmission dynamics of SARS-CoV-2 through the postpandemic period. Science..

[18] Giordano G, Blanchini F, Bruno R, Colaneri P, Di Filippo A, Di Matteo A (2020). Modelling the COVID-19 epidemic and implementation of population-wide interventions in Italy. Nat Med..

[19] Belot M, Choi S, Jamison JC, Papageorge NW, Tripodi E, van den Broek-Altenburg E. Six-country survey on COVID-19. Work Paper Database. 2020; https://osf.io/aubkc/.

[20] Clark N, Becker M, Shumaker S, Schron E, Ockene J, McBee W (1998). Theoretical models and strategies for improving adherence and disease management. The handbook of health behavior change.

[21] Leung GM, Ho LM, Chan SK, Ho SY, Bacon-Shone J, Choy RY (2005). Longitudinal assessment of community psychobehavioral responses during and after the 2003 outbreak of severe acute respiratory syndrome in Hong Kong. Clin Infect Dis..

[22] Laaksonen M, Prättälä R, Helasoja V, Uutela A, Lahelma E (2003). Income and health behaviours. Evidence from monitoring surveys among Finnish adults. J Epidemiol Community Health.

[23] Rubin GJ, Amlôt R, Page L, Wessely S (2009). Public perceptions, anxiety, and behaviour change in relation to the swine flu outbreak: cross sectional telephone survey. BMJ.

[24] Wolf MS, Serper M, Opsasnick L, O'Conor RM, Curtis L, Benavente JY (2020). Awareness, attitudes, and actions related to COVID-19 among adults with chronic conditions at the onset of the U.S. outbreak: a cross-sectional survey. Ann Intern Med.

[25] Des Jarlais DC, Galea S, Tracy M, Tross S, Vlahov D (2006). Stigmatization of newly emerging infectious diseases: AIDS and SARS. Am J Public Health.

[26] Cialdini RB, Goldstein NJ (2004). Social influence: compliance and conformity. Annu Rev Psychol.

[27] Abrams D, Wetherell M, Cochrane S, Hogg MA, Turner JC (1990). Knowing what to think by knowing who you are: self-categorization and the nature of norm formation, conformity and group polarization. Br J Soc Psychol.

