# Supplementary material for: Public perceptions, individual characteristics, and preventive behaviors for COVID-19 in six countries: a cross-sectional study
Source: Environ Health Prev Med. 2021 Mar 3;26:29. doi: 10.1186/s12199-021-00952-2 (PMC7928175; doi:10.1186/s12199-021-00952-2)
Supplement: Supplementary file 1 — Additional file 1. The online self-administered questionnaire for this six-country survey. This questionnaire was used to assess individual perceptions of COVID-19, and their behavioral change. [file 12199_2021_952_MOESM1_ESM.pdf]

currently using.

This study was cleared by the ethics committee of the University of Exeter. Should you have any question, difficulty, or complaint, please contact Prof. Julian Jamison at J.Jamison@exeter.ac.uk.

By clicking on “Yes, I would like to participate in the survey” you give your consent to take part in the study and the survey will be launched.

- Yes, I would like to participate in the survey
- No, I would not like to participate in the survey

Socio-demographics

How old are you?

- Below 18
- Between 18 and 25
- Between 26 and 35
- Between 36 and 45
- Between 46 and 55
- Between 56 and 65
- Between 66 and 75
- Above 75
- Prefer not to answer

What is your gender?

Male	Female	Prefer not to answer
○	○	○

What is your race (select all that apply)?

- ☐ American Indian or Alaskan Native
- ☐ Asian
- ☐ Black or African American
- ☐ Native Hawaiian or Other Pacific Islander
- ☐ White / Caucasian
- ☐ Prefer not to answer

What state do you live in?

Select state

What is your work situation

- ○ Self-employed
- ○ Employed part-time
- ○ Employed full-time
- ○ Not in employment

In what range is the gross annual income of your household?

- ○ $23,000 or less
- ○ $23,001 - $42,000
- ○ $42,001 - $66,000
- ○ $66,001 - $106,000
- ○ $106,001 or more
- ○ Prefer not to answer

These page timer metrics will not be displayed to the recipient.
First Click: *0 seconds*

Last Click: *0 seconds*
Page Submit: *0 seconds*
Click Count: *0 clicks*

General health status and risk factors

Some of the next questions are denoted by an “*” to indicate that they ask information that may be more personal in nature and will be kept confidential.

Do you have any of the following conditions? (select all that apply)*

- ☐ Diabetes
- ☐ High blood pressure / hypertension
- ☐ Heart disease
- ☐ Asthma or other chronic respiratory issues
- ☐ Allergies
- ☐ Other chronic illnesses that require long term care from a doctor (please specify) ______
- ☐ None of the above

Are you vaccinated against influenza this season?

Yes	No
○	○

Are you a smoker?

Yes	No
○	○

Have you in the past two weeks experienced any of the following symptoms?
*

- ☐ Dry cough
- ☐ Fever
- ☐ Tiredness
- ☐ Runny nose
- ☐ Sore throat
- ☐ Nasal congestion
- ☐ Aches and pains
- ☐ Diarrhea
- ☐ Loss of smell or taste
- ☐ [ ] Any other symptoms
- ☐ None of the above

These page timer metrics will not be displayed to the recipient.
First Click: *0 seconds*
Last Click: *0 seconds*
Page Submit: *0 seconds*
Click Count: *0 clicks*

When did you first experience any symptom?*

	Month	Day	Year
Please Select:			2020

Have you been in contact with your doctor or the health authorities?

- ○ Yes, and they said that getting tested for Covid-19 at this stage is not necessary
- ○ Yes, and I am waiting to be tested

- No, I don't think my symptoms are serious enough to require medical attention
- Other

Exposure -- Part 1

How many people that you have interacted with recently have been diagnosed with Covid 19?

On a typical working day (before the outbreak of Covid-19), with how many people would you have close social contact with (at less than 1 metre distance) and how long would you interact with them? (indicate approximate numbers - leave blank if the answer is zero)

	Children (18 or younger)	Adults (between 19 and 60)	Adults (older than 60)
For less than 15 minutes			
For more than 15 minutes			

What is your profession?

Industry

Profession

In what type of area are you currently living?

Urban area	Semi-urban / residential	Countryside

My current home is best described as

- House
- Apartment
- Condominium
- Trailer
- Shelter
- Other (please specify)

Who shares the kitchen or other facilities in your accommodation? Please indicate the number of people who are:

	leave blank if 0
Under the age of 2	
Between the age of 2 and 12	
Between the age of 13 and 18	
Between the age of 19 and 25	
Between the age of 26 and 50	
Between the age of 50 and 65	
Above 65	

Which of the following best describes your current living situation?

- Live alone in my home (may have a pet)

- Live in a household with other people
- Live in a facility such as a nursing home which provides meals and 24-hour nursing care
- Temporarily staying with a relative or friend
- Temporarily staying in a shelter or homeless

What outside space do you have access to in your current accommodation? (select all that apply)

- None
- Balcony
- Terrace
- Small private garden or Yard (less than 200 sq ft)
- Large private garden or Yard (more than 200 sq ft)
- Common courtyard
- Other

These page timer metrics will not be displayed to the recipient.
First Click: *0 seconds*
Last Click: *0 seconds*
Page Submit: *0 seconds*
Click Count: *0 clicks*

Exposure -- Part 2

In normal times (before the outbreak of Covid 19), how often would you use the following means of transportation over the course of two weeks?

	Never	Once	Twice	More than twice
Car	○	○	○	○

Bus / metro / tram / train (< 2 hours) outside rush hours	○	○	○	○
Bus / metro / tram / train (< 2 hours) during rush hours	○	○	○	○
Long journey coach / train (> 2 hours)	○	○	○	○
Plane	○	○	○	○

Have you travelled abroad or outside your local area since January 2020?

Yes	No
○	○

Please indicate if you have been in any of the following areas (select all that apply):

- ☐ Wuhan
- ☐ Other regions in China
- ☐ North of Italy (Lombardia, Veneto, Emilia Romagna)
- ☐ Other regions in Italy
- ☐ ______ Other region/country (please indicate)

If you have stayed outside your home, have you stayed in facilities which have shared facilities with other people (such as hotels, ski resorts, cruiseships)?

Yes	No
○	○

Behavioral response

Due to the Covid-19 epidemic, have you changed any of your daily behaviors?

Yes	No
○	○

What caused your behavior to change (select all that apply)?

- ☐ To protect myself
- ☐ To protect my family
- ☐ To protect the public (people I don't know)
- ☐ Because everyone else did
- ☐ Due to recommendations from friends/family
- ☐ Due to recommendations from doctors or public health officials
- ☐ Due to recommendations from politicians
- ☐ Contact with people infected with Covid-19
- ☐ Appearance of Covid-19-related symptoms
- ☐ Diagnosis of Covid-19
- ☐ Legal measures (e.g. a stay at home order)
- ☐ ______ Other (please specify)

For each of the following behaviors, how often do you engaged in them since the Covid 19 outbreak first started spreading in your country?

	Never	Rarely	Sometimes	Very often	Always
Keeping at least 4 ft distance from anyone who is coughing or sneezing	○	○	○	○	○

Avoiding touching eyes, nose and mouth	○	○	○	○	○
Covering your mouth and nose with your bent elbow or tissue when you cough or sneeze. Then dispose of the used tissue immediately	○	○	○	○	○
If you have fever, cough and difficulty breathing, seek medical care early	○	○	○	○	○

For each of the following behaviors, please tell us how often you engaged in them at different times

	In normal times (before the outbreak of Covid-19)	Soon after the Covid 19 outbreak started in your country	Now
Wash your hands with water and soap, or use hand sanitizer		Same as in "normal times"	Same as in "since out
Wear a mask		Same as in "normal times"	Same as in "since out
Eat at least 5 portions of fruit and vegetables a day		Same as in "normal times"	Same as in "since out
Exercise		Same as in "normal times"	Same as in "since out

Meet friends/family through video chat		Same as in "normal times"	Same as in "since out
Smoke		Same as in "normal times"	Same as in "since out
Take vitamins or supplements		Same as in "normal times"	Same as in "since out
Participate in a social gathering with more than 20 people		Same as in "normal times"	Same as in "since out
Go to a large closed space such as a museum, shopping malls or supermarkets		Same as in "normal times"	Same as in "since out
Go to a large open space such as a public park		Same as in "normal times"	Same as in "since out
Run errands such as filling petrol, going to the bank or post office, buying items in small shops		Same as in "normal times"	Same as in "since out
Visit family or friends		Same as in "normal times"	Same as in "since out
Attend a GP practice		Same as in "normal times"	Same as in "since out
Use public transport		Same as in "normal times"	Same as in "since out

Order meal deliveries		Same as in "normal times"	Same as in "since out

These page timer metrics will not be displayed to the recipient.

First Click: *0 seconds*

Last Click: *0 seconds*

Page Submit: *0 seconds*

Click Count: *0 clicks*

How did your work situation change in the recents weeks as a consequence of the pandemic?

I do not work anymore	I started teleworking	No change	Other	Not applicable
◯	◯	◯	◯	◯

Have you moved accommodation or location as a consequence of the epidemic?

Yes	No
◯	◯

In what type of area are you currently living?

- ◯ Urban area
- ◯ Semi-urban / residential
- ◯ Countryside

On a typical day in the last 2 weeks, how many close contacts (less than 4 ft distance) have you had and with whom? (indicate approximate numbers - leave blank if the answer is zero)

	Children (18 or younger)	Adults (between 19 and 60)	Adults (older than 60)
For less than 15 minutes			
For more than 15 minutes			

Over the past two weeks, to what extent have you managed to reduce in person interactions in your work context?

Not at all	Partly	To a large extent	Completely	Not applicable
○	○	○	○	○

With what weekly frequency have you done any of the following activities since the Covid 19 outbreak in your country?

	Not at all	At least once	Almost every day	Every day
Bought groceries or medicines for friends or family in quarantine	○	○	○	○
Bought groceries or medicines for friends or family members in critical risk groups (e.g. elderly immunocompromised)	○	○	○	○
Other volunteer activities	○	○	○	○
Attended religious services	○	○	○	○

Have you had to defer needed health care because of the Covid 19 outbreak and related measures?

Yes ○ No ○

Was the deferred care for:

- ○ Routine preventive services
- ○ Ongoing care for a continuing health problem
- ○ Care for a newly developed problem

How concerned are you that the deferred care is going to harm your health?

Extremely concerned	Very concerned	Somewhat concerned	Slightly concerned	Not at all concerned
○	○	○	○	○

Have you ever used telemedicine for healthcare?

Yes ○ No ○

Would you consider consider using telemedicine for your deferred healthcare?

Yes ○ No ○

Individual economic and mental consequences of the virus

How well can you work from home relative to your normal work situation?

Not at all	Not well	Somewhat well	Quite well	Essentially unaffected

Have you lost your job or has your actitivity (as self-employed) been stopped as a consequence of the Covid-19 pandemic?

Yes, permanently	Yes, temporarily	No
○	○	○

How has your workload changed as a consequence of the Covid 19 pandemic?

I work more hours	I work the same number of hours as before	I work less hours
○	○	○

Have you experienced a fall in household income as a consequence of the Covid-19 pandemic?

Yes	No
○	○

How much did your gross household income fall in the first trimester of 2020? (provide estimate in USD)

Do you expect substantial loss in the labor income of your household (including self-employment income) as a consequence of the Covid 19 pandemic for the six months from April to September of 2020?

Yes	No
○	○

How much do you expect

	provide estimate in USD
Your own gross labor income to fall in the next six months?	
Your gross household income to fall in the next six months?	

Do you currently have a mortgage on your house?

Yes	No
○	○

How did your weekly expenses change relative to the month of January?

- Drop of more than 10%
- Drop of less than 10%
- No change
- Increase of less than 10%
- Increase of more than 10%

How did your savings change relative to the month of January?

- Drop of more than 10%
- Drop of less than 10%
- No change
- Increase of less than 10%
- Increase of more than 10%

Beliefs and preferences for interventions

If restrictions to stop the pandemic are currently in place in your country, how bothered are you for not being able to do the following activities?

	Not at all bothered	Slightly bothered	Moderately bothered	Very bothered	Extreme bothere
Working at your usual workplace	○	○	○	○	○
Meeting other people in your leisure time	○	○	○	○	○
Engage in leisure activities outside your home (e.g. sport, restaurants, ...)	○	○	○	○	○
Go into non-essential shops	○	○	○	○	○
Travelling for work	○	○	○	○	○
Travelling for leisure	○	○	○	○	○
Anything else [ ]	○	○	○	○	○

How effective do you believe each of these measures is in reducing the spread of the epidemic?

	Not effective at all	Slightly effective	Moderately effective	Very effective	Extremely effective
Shutting down schools	○	○	○	○	○
Shutting down public transport	○	○	○	○	○

Shutting down non-essential businesses	○	○	○	○	○
Limiting mobility outside home	○	○	○	○	○
Forbidding mass gatherings	○	○	○	○	○
Introducing fines for citizens that don't respect public safety measures	○	○	○	○	○
Requiring masks to be worn outside by everyone	○	○	○	○	○

How effective do you believe hand washing is in reducing the spread of the epidemic?

Not effective at all	Slightly effective	Moderately effective	Very effective	Extremely effective
○	○	○	○	○

What fraction of people in your local area do you think...?

Fraction in percentage points %

0 5 10 15 20 25 30 35 40 45 50 55 60 65 70 75 80 85 90 95 100

Are currently infected?

What do you think is the probability that...

Probability in percentage points %

0 5 10 15 20 25 30 35 40 45 50 55 60 65 70 75 80 85 90 95 100

You are or have been infected with Covid 19?

What do you think is the probability that an infected person...?

	Probability in percentage points %
	0 5 10 15 20 25 30 35 40 45 50 55 60 65 70 75 80 85 90 95 100
Develops no symptoms	
Develops a serious illness (stay in bed) without requiring hospitalization	
Develops a serious illness that requires hospitalization	
Dies	

Have you experienced any positive non-financial effects from the societal changes occurring due to the epidemic, such as (select all that apply):

- ☐ Enjoying more free time
- ☐ Enjoying time with family
- ☐ Reduction of air pollution
- ☐ Reduction of noise pollution
- ☐ ________ Other
- ☐ None of the above

Have you experienced any negative non-financial effects from the societal changes occurring due to the epidemic, such as (select all that apply):

- ☐ Boredom

- ☐ Loneliness
- ☐ Trouble sleeping
- ☐ General anxiety and stress
- ☐ Increased conflicts with friends, relatives and neighbours
- ☐ ________ Other
- ☐ None of the above

How happy do you feel these days?

Extremely unhappy	Moderately unhappy	Slightly unhappy	Neither happy nor unhappy	Slightly happy	Moderately happy	Extremely happy
○	○	○	○	○	○	○

Do you agree with the current approach taken by your government in response to the pandemic?

Strongly disagree	Somewhat disagree	Neither agree nor disagree	Somewhat agree	Strongly agree
○	○	○	○	○

In comparison to a regular influenza epidemic you have experienced in the last 5 years and in the absence of any measures for Covid 19, do you think the health consequences (number of infected people and number deaths) of the Covid 19 epidemic would be?

Much lower	Lower	About the same	Higher	Much higher
○	○	○	○	○

These page timer metrics will not be displayed to the recipient.

First Click: *0 seconds*

Last Click: *0 seconds*

Page Submit: *0 seconds*

Click Count: *0 clicks*

Powered by Qualtrics
